# Xenobiotics Triggering Acute Intermittent Porphyria and Their Effect on Mouse Brain Respiratory Complexes †

**DOI:** 10.3390/jox14010019

**Published:** 2024-02-27

**Authors:** Johanna Romina Zuccoli, María del Carmen Martínez, Pablo Vallecorsa, Ana María Buzaleh

**Affiliations:** 1 Research Center on Porphyrins and Porphyrias (CIPYP)—UBA-CONICET, Hospital of Clinics José de San Martín, Buenos Aires 1120, Argentina; johannazuccoli@gmail.com (J.R.Z.); mcmartin@qb.fcen.uba.ar (M.d.C.M.); 2 Department of Biological Chemistry, Faculty of Exact and Natural Sciences, University of Buenos Aires, Buenos Aires 1428, Argentina; 3 National Atomic Energy Commission (CNEA), Buenos Aires 1429, Argentina; pvallecorsa@yahoo.com

**Keywords:** mitochondria, respiratory chain complexes, isoflurane, sevoflurane, porphyrinogenic agents, acute intermittent porphyria, 5-aminolevulinic acid

## Abstract

Heme enzyme dysfunction causes a group of diseases called porphyrias. Particularly, a decrease in porphobilinogen deaminase, involved in the third step of heme biosynthesis, leads to acute intermittent porphyria (AIP). Considering our previous works demonstrating the multiplicity of brain metabolisms affected by porphyrinogenic agents, this study aimed to elucidate whether they cause any alteration on the mitochondrial respiratory chain. The activities of respiratory chain complexes (I to IV) were measured in encephalon mitochondria of *CF1* male mice receiving volatile anesthetics: isoflurane (2 mL/kg) and sevoflurane (1.5 mL/kg), ethanol (30%), allylisopropylacetamide (AIA) (350 mg/kg), and barbital (167 mg/kg). Moreover, they were compared versus animals with pathological levels of 5-aminolevulinic acid (ALA, 40 mg/kg). Complex I–III activity was induced by isoflurane and decreased by AIA, ethanol, and ALA. Complex II–III activity was increased by sevoflurane and decreased by isoflurane and AIA. Complex II activity was increased by sevoflurane and barbital and decreased by AIA, ethanol, and ALA. Complex IV activity was increased by barbital and ALA and decreased by sevoflurane. The damage to the respiratory chain by ALA could be reflecting the pathophysiological condition of patients with AIP. Better understanding the broad effect of porphyrinogenic drugs and the mechanisms acting on the onset of AIP is vital in translational medicine.

## 1. Introduction

Disorders in heme biosynthesis leads to a group of diseases called porphyrias [1,2,3]. In particular, a decrease in porphobilinogen deaminase (PBG-D), the enzyme involved in the third step of heme biosynthesis, leads to acute intermittent porphyria (AIP) [4,5,6]. This hepatic porphyria is characterized by neurological symptoms that consist of central nervous system dysfunction and motor polyneuropathy. Neurological damage and axonal degeneration can be primary pathological lesions with secondary demyelination [7,8]. These manifestations could be due to the accumulation of 5-aminolevulinic acid (ALA), which is synthesized by 5-aminolevulinic acid synthase (ALA-S), the regulatory enzyme of the heme pathway [9,10,11,12]. Moreover, the acute attacks observed in AIP could be a consequence of a reduced availability of heme for hemeproteins [13]. The decrease in heme due to failures in its biosynthesis or by an accelerated catabolism causes mitochondrial membrane potential loss, oxidative stress, calcium homeostasis alterations, and the exit of cytochrome c from the mitochondria [14].

The onset of acute attacks in AIP is due to exogenous factors such as drugs, in addition to fasting and stress [15,16]. A high percentage of individuals with AIP develop a crisis after or during a surgery [17].

Sevoflurane, the most-used anesthetic, is used for the inhalational induction of general anesthesia due to its low blood–gas solubility and rapid onset, but this drug is potentially neurotoxic [18,19]. In young rats, sevoflurane induces neuroapoptosis via several mechanisms, generating mitochondrial outer membrane permeabilization and inducing intrinsic apoptosis [19,20]. In rat and human synaptosomes, sevoflurane depolarizes mitochondria via the inhibition of chain respiratory ATP synthase [21]. Isoflurane, a volatile anesthetic of the same family as sevoflurane [22], affects Complex I activity and reduces ATP synthesis. Although both anesthetics cause mitochondrial toxicity, in vitro studies have revealed that sevoflurane is less active than isoflurane [23].

Isoflurane and sevoflurane reproduced the typical biochemical signs of AIP when they were administered to *CF1* mice [24,25,26] or to the AIP mice model [27]. ALA-S and PBG-D activities were altered depending on the anesthetic, the mice strain study, and the tissue analyzed.

In *CF1* mice brains, we have demonstrated that the administration of volatile anesthetics and other known porphyrinogenic drugs leads to alterations in the cholinergic and glutamatergic system [28,29], the antioxidant defense system [25], the phase I drug metabolizing system [30,31], and the synthesis of nitric oxide (NO) [32]. In addition, previous studies have shown that ALA administration to *CF1* mice also causes alterations in numerous metabolic pathways in the brain of mice [33].

In AIP subjects, mitochondrial function alterations were first reported by Bonkovsky et al. [34], who found alterations in mitochondrial NADH oxidation in cultured skin fibroblasts. Then, Dixon et al. [35] observed a limited electron transport and ATP synthesis in patients with AIP which have a decrease in their bioenergetics capacity, and a lower oxygen consumption rate was less prevalent in those patients with a manifested disease.

Several metabolic processes, such as oxidative phosphorylation, Krebs cycle, fatty acid β-oxidation, calcium handling, and some steps of heme biosynthesis, take place in mitochondria [36,37,38]. Some key proteins of the oxidative phosphorylation like ubiquinol-cytochrome c oxide reductase (Complex III), cytochrome oxidase (Complex IV), and cytochrome c (which participates transporting electrons from Complex III to Complex IV) are hemeproteins [39,40]. Thus, mitochondrial malfunctioning has been related to metabolic alterations, cancer, neurodegenerative syndromes, and aging [36,37,41,42,43,44].

Considering the widespread effects of porphyrinogenic drugs and ALA observed in our previous works, the objective of this study was to explore the action of these drugs on the mitochondrial respiratory chain. For this purpose, the effects of volatile anesthetics such as isoflurane and sevoflurane, allylisopropylacetamide (AIA), barbital, and ethanol were evaluated in mouse brain mitochondria. A comparison was made with mice under pathological levels of ALA. The activities of NADH-cytochrome c reductase (Complex I–III, EC 1.6.5.3 + 1.10.2.2), succinate-cytochrome c reductase (Complex II–III, EC 1.3.5.1 + 1.10.2.2), succinate dehydrogenase (Complex II, EC 1.3.5.1), and cytochrome c oxidase (Complex IV, EC 1.9.3.1) were measured, and NADH/ubiquinone oxidoreductase (Complex I, EC 1.6.5.3) activity was estimated using the relationship between the activities of Complexes I–III and II–III.

## 2. Materials and Methods

### 2.1. Chemicals

All the chemicals used were reagent grade (Sigma Chem. Co., St. Louis, MI, USA). Isoflurane and sevoflurane were from Abbott Laboratories S.A. (Abbott Park, IL, USA).

### 2.2. Animals

Animals were treated following the rules established by the Animal Care and Use Committee of the Argentine Association of Specialists in Laboratory Animals (AADEALC) and housed under controlled conditions and free access to food (Purina 3, Asociación de Cooperativas Argentinas, San Nicolás, Buenos Aires, Argentina) and water. The project was approved by the Institutional Committee for Use and Care of Laboratory Animals (CICUAL), School of Medicine, University of Buenos Aires, Argentina (Res. 2371; 13 October 2017).

For all the experiments, control mice (untreated) and treated mice (4–6 animals/group), *CF1* strain (*crlfcen: CF1*, outbred), were used.

### 2.3. Treatments

The experimental design was based on our previous work [30,33]. Particularly, the protocols for isoflurane and sevoflurane were supported by our earlier research [24,26].

The doses and time of administration of each drug were as follows: Animals received a single dose intraperitoneal (i.p.) of isoflurane (2 mL/kg) or sevoflurane (1.5 mL/kg) and were then euthanized 20 min after the injection. In the case of AIA, a single dose of 350 mg/kg (i.p.) 16 h prior to euthanasia was injected. For barbital, mice received three doses (one dose of 167 mg/kg, subcutaneous (s.c.) for 3 days), and were then euthanized 24 h after the last dose. Ethanol (30%, *v*/*v*) was administered in drinking water for a week. The mice that received ALA were injected with one dose (40 mg/kg, i.p.) 24 h prior to euthanasia. In all the cases, the corresponding control groups only received a vehicle (oil for isoflurane and sevoflurane; 0.9% NaCl/ethanol (1:3 *v*/*v*) for AIA; water for ethanol, and 0.9% NaCl for barbital and ALA) and were euthanized at the times established for each drug. Euthanasia was performed by exposure to carbon dioxide.

### 2.4. Preparation of Subcellular Fractions and Assays

Homogenates of whole brain, performed in potassium phosphate buffer pH 7.2 plus 0.15 M NaCl, were centrifuged at 700× *g* for 10 min and then at 8000× *g* for 10 min to obtain the crude mitochondrial fraction. The pellet was washed once with the same solution used to prepare the homogenate, resuspended in mannitol buffer plus 6% Ficoll solution, and centrifuged again (12,500× *g*, 30 min). This new pellet was resuspended in 0.23 M mannitol, 0.07 M sucrose, and 30 mM buffer (Tris-HCl pH 7.5). Cytochrome was measured to assess mitochondrial purity [45].

The activities of NADH-cytochrome c reductase (Complexes I–III) and succinate-cytochrome *c* reductase (Complexes II–III) were determined using NADH or succinate as substrates, respectively, following the reduction of cytochrome *c*^3+^ [46] at 550 nm (ε = 19.6 mM^−1^·cm^−1^) for 2 min at 37 °C. The incubation system to determine Complexes I–III contained: 0.1 M potassium phosphate buffer pH 7.4, 0.2 mM NADH, 0.5 mM KCN, cytochrome c^+3^ 25 µM, and mitochondrial fraction (0.02 mg/mL protein), in a final volume of 1 mL. A blank reaction without enzyme was included. When Complexes II–III were measured, the incubation system was the same, but 5 mM succinate was added instead of NADH. One enzyme unit (U) was defined as the amount of enzyme that catalyzes the synthesis of 1 nmol of reduced cytochrome c per minute under standard incubation conditions.

Succinate dehydrogenase (Complex II) activity was quantified monitoring 2,6-dichlorophenolindophenol (DCIP) reduction at 600 nm (ε = 21 mM^−1^·cm^−1^) for 2 min at 37 °C, as stated by King [47]. The incubation system contained the following in a final volume of 1 mL: 0.2 M buffer potassium phosphate pH 7.4, 0.6 M succinate, 3 mM EDTA, 0.53 mM DCIP, and mitochondrial enzyme fraction (0.02 mg/mL). A blank reaction without an enzyme was included. One U was defined as the amount of enzyme that catalyzes the formation of 1 nmol of reduced DCIP per minute under standard incubation conditions.

To estimate Complex I (NADH/ubiquinone oxidoreductase) activity, the ratio between the activities of Complexes I–III and II–III was calculated for each sample to provide an internal standard for qualitative Complex I activity analysis [48].

To measure cytochrome c oxidase (Complex IV) activity, the decrease in the absorbance at 550 nm of reduced cytochrome c^+2^ was followed at 550 nm (ε = 39.4 mM^−1^·cm^−1^) for 1 min at 37 °C, as reported by Yonetani et al. [49]. The incubation system contained the following in a final volume of 1 mL: 0.05 M sodium phosphate buffer pH 7.4, 50 µM cytochrome c^+2^ and mitochondrial enzyme fraction (0.02 mg/mL). A reaction blank without an enzyme was included. One U was defined as the amount of enzyme that catalyzes the oxidation of 1 nmol of cytochrome c per minute under standard incubation conditions.

Protein concentration was measured using the technique of Lowry et al. [50].

### 2.5. Statistical Analysis

Data are expressed as percentage respect to control values of the day on which the experiments were conducted. The mean and standard deviation (s.d.) of each group variable were calculated and compared between treated and untreated cohorts using analysis of variance (ANOVA) (Excel 365 program, Microsoft); a probability level less than 0.05 was considered significantly different.

## 3. Results

### 3.1. Alterations in the Mitochondrial Respiratory Chain

To investigate the effect of some porphyrinogenic drugs, such as isoflurane, sevoflurane, AIA, barbital, and ethanol on the brain respiratory chain, the activities of Complexes I to IV were measured in brain mitochondria. The action of ALA was also analyzed.

#### 3.1.1. Effect of Porphyrinogenic Drugs

**Figure 1:**
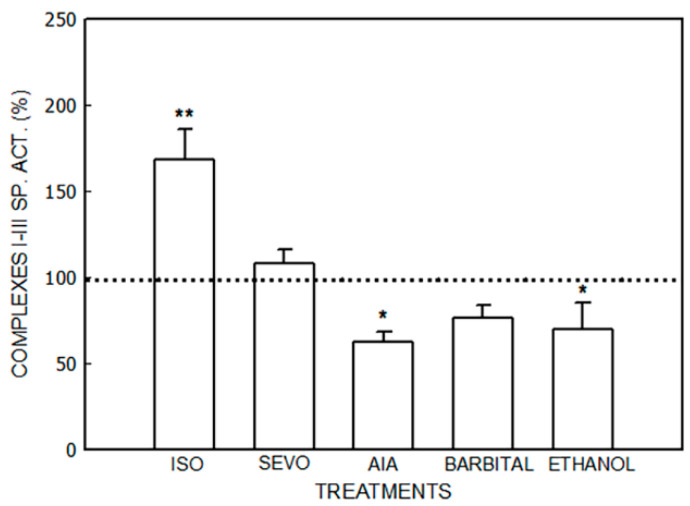
Activity of NADH-cytochrome C reductase (Complexes I–III) after administration of isoflurane (ISO), sevoflurane (SEVO), allylisopropylacetamide (AIA), barbital and ethanol to mice. Doses of drugs were isoflurane (2 mL/kg, i.p.), sevoflurane (1.5 mL/kg, i.p.), AIA (350 mg/kg, i.p.), barbital (167 mg/kg, s.c), and ethanol (30%, *v*/*v*). Data represent mean value ± S.D. of 4–6 animals and are expressed as a percentage considering the daily control group as 100% (dotted line). * *p* < 0.05, ** *p* < 0.01: significance of differences between the treated and control groups. A single control value is given because no significant differences were obtained in any of the controls after the administration of the vehicle. Control value (nmol/mg): 55.9 ± 17.7. Other experimental details are described in the Section 2.

**Figure 2:**
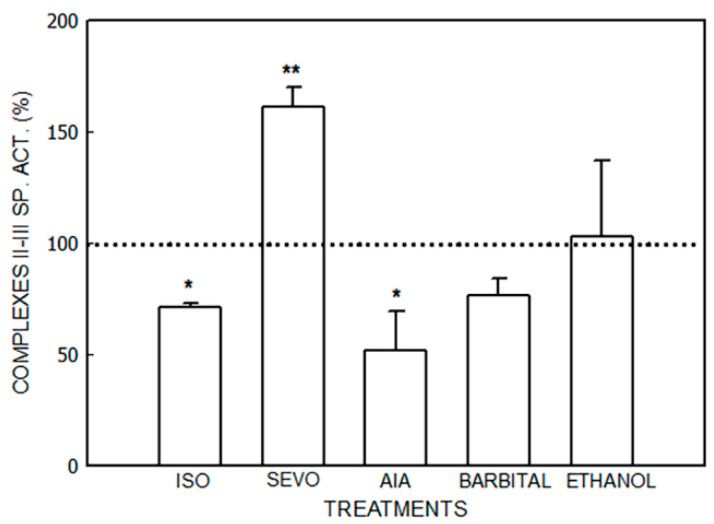
Activity of succinate-cytochrome C reductase (Complexes II–III) after administration of isoflurane (ISO), sevoflurane (SEVO), allylisopropylacetamide (AIA), barbital and ethanol to mice. Data represent mean value ± S.D. of 4–6 animals and are expressed as a percentage considering the daily control group as 100% (dotted line). * *p* < 0.05, ** *p* < 0.01: significance of differences between the treated and control groups. A single control value is given because no significant differences were obtained in any of the controls after the administration of the vehicle. Control value (nmol/mg): 16.3 ± 5.9. Other experimental details are described in the legend of Figure 1 and in the Section 2.

**Figure 3:**
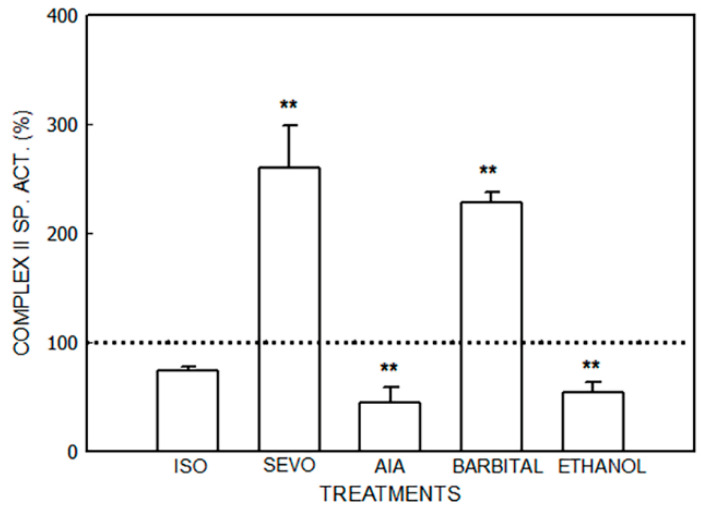
Activity of succinate dehydrogenase (Complex II) after administration of isoflurane (ISO), sevoflurane (SEVO), allylisopropylacetamide (AIA), barbital and ethanol to mice. Data represent mean value ± S.D. of 4–6 animals and are expressed as a percentage considering the daily control group as 100% (dotted line). ** *p* < 0.01: significance of differences between the treated and control groups. A single control value is given because no significant differences were obtained in any of the controls after the administration of the vehicle. Control value (nmol/mg): 70.7 ± 13.0. Other experimental details are described in the legend of Figure 1 and in the Section 2.

**Figure 4:**
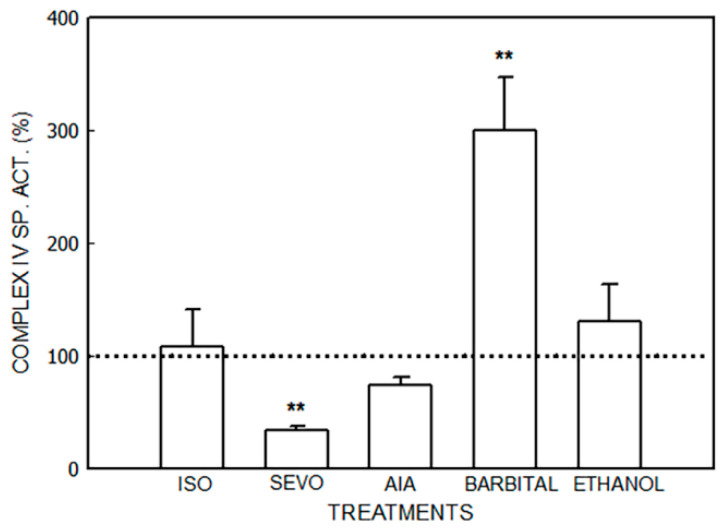
Activity of cytochrome c oxidase (Complex IV) after administration of isoflurane (ISO), sevoflurane (SEVO), allylisopropylacetamide (AIA), barbital and ethanol to mice. Data represent mean value ± S.D. of 4–6 animals and are expressed as a percentage considering the daily control group as 100% (dotted line). ** *p* < 0.01: significance of differences between treated and control groups. A single control value is given, because no significant differences were obtained in any of the controls after the administration of the vehicle. Control value (nmol/mg): 4.2 ± 1.6. Other experimental details are described in the legend of Figure 1 and in the Section 2.

The effects of isoflurane, sevoflurane, AIA, barbital, and ethanol are shown in Figure 1, Figure 2, Figure 3 and Figure 4 and Table 1.

NADH-cytochrome c reductase (Complexes I–III) activity (Figure 1) was increased by 69% (*p* < 0.01) via the treatment with isoflurane and decreased by 38% by AIA (*p* < 0.05) and by 30% by ethanol (*p* < 0.05). No significant changes were observed after sevoflurane or barbital administration.

Succinate-cytochrome c reductase (Complexes II–III) activity (Figure 2) was increased by 61.5% by sevoflurane (*p* < 0.01) and decreased by 30% by isoflurane (*p* < 0.05) and by 49% by AIA (*p* < 0.01). No variations were observed in the animals that received barbital or ethanol.

Succinate dehydrogenase (Complex II) activity (Figure 3) was increased by 160% by sevoflurane (*p* < 0.01) and by 127% by barbital (*p* < 0.01), and decreased by 51% by AIA (*p* < 0.01) and by 46% by ethanol (*p* < 0.01). No variations were observed in the animals that received isoflurane.

Cytochrome c oxidase (Complex IV) activity (Figure 4) was increased by 200% by barbital (*p* < 0.01) and decreased by 77% by sevoflurane (*p* < 0.01). No variations were observed in the animals that received isoflurane, AIA, or ethanol.

The calculation of the ratio between Complexes I–III and II–III to estimate alterations in NADH/ubiquinone oxidoreductase (Complex I) activity showed, in general, a greater activity of Complex I than Complex II (Table 1). When the comparison was made between the treated and control groups, the lower ratio in groups receiving isoflurane, sevoflurane, barbital, and AIA indicated a reduction in the activity of Complex I. A contrary effect was caused by ethanol administration, which led to an increase in the activity of Complex I.

#### 3.1.2. Effect of ALA

**Figure 5:**
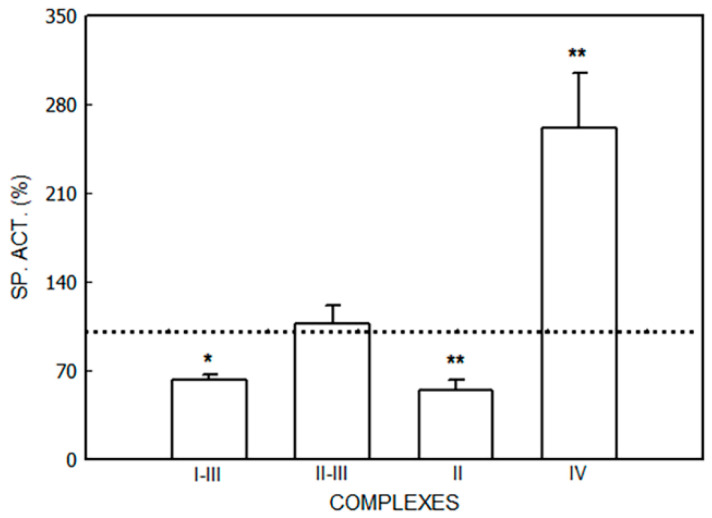
Activity of respiratory chain complexes (Complexes I–IV) after ALA administration to mice. Animals received ALA (40 mg/kg, i.p.). Data represent mean value ± S.D. of 4–6 animals and are expressed as a percentage considering the daily control group as 100% (dotted line). * *p* < 0.05, ** *p* < 0.01: significance of differences between the treated and control groups. A single control value is given because no significant differences were obtained in any of the controls after the administration of the vehicle. Control values (nmol/mg): Complexes I–III: 55.9 ± 17.7, Complexes II–III: 16.3 ± 5.9, Complex II: 70.7 ± 13.0, Complex IV: 4.2 ± 1.6. Other experimental details are described in the Section 2.

The effects of ALA on the respiratory chain complexes are shown in Figure 5.

A decrease was detected in NADH-cytochrome c reductase (Complexes I–III) activity (37%, *p* < 0.05). No alterations were found in succinate-cytochrome c reductase (Complexes II–III). The rate between these complexes (I–III/II–III) was higher in ALA-treated mice (3.32 ± 0.44) than in non-treated ones (5.31 ± 1.24), indicating a reduction in Complex I activity. Succinate reductase (Complex II) activity was decreased by ALA (55%, *p* < 0.01), whereas cytochrome c oxidase (Complex IV) activity was increased (161%, *p* < 0.01).

## 4. Discussion

The effects of volatile anesthetics and other porphyrinogenic agents on the brain respiratory chain of *CF1* male mice were investigated. The activities of all the complexes were affected, and the magnitude of the alteration varied according to the agent studied. Briefly, isoflurane increased Complex I–III and decreased Complex II–III activities. Sevoflurane increased Complexes II and II–III and decreased Complex IV activities. AIA decreased the activities of Complexes I–III, II, and II–III. Barbital increased Complex II and IV activities. Ethanol only generated biochemical alterations in both Complex II and Complex I–III. Under pathophysiological conditions of high concentrations of ALA, the activities of Complexes I–III and II were decreased, whereas the activity of Complex IV was increased.

It has been proposed that respiratory complexes are linked among them, forming structures called super complexes, which make their function more effective [51,52]. Vikramdeo et al. [52] summarized the data on mitochondrial syndromes associated with alterations in the activity of each complex. Moreover, it is important to mention that mitochondrial integrity and functionality is crucial for brain energy maintenance [53].

The alterations reported here regarding respiratory complexes would lead to a disruption in mitochondrial function, also generating oxidative damage, which could be a consequence of the alterations in heme metabolism caused by the xenobiotics evaluated. Complex I is very important because it is the first entry point of electrons into the respiratory chain [54,55]. Thus, its deficiency could be one of the most common factors leading to mitochondrial alterations [56]. In agreement with this, it has been reported that the activity of Complex I is decreased in individuals with Parkinson’s disease [48,57].

In vitro, both isoflurane and sevoflurane cause the depolarization of the mitochondrial membrane via the inhibition of Complex I [23,58,59]. Mitochondrial respiration can be impaired by sevoflurane exposure, which leads to an enhanced production of reactive oxygen species (ROS), impaired mitochondrial membrane permeabilization, and reduced ATP production, possibly with subsequent cell damage and apoptosis [60].

In our work, isoflurane and sevoflurane affected the respiratory chain in vivo, also reducing Complex I activity. The deficiency in Complex I probably occurs secondarily to the oxidative stress injury previously described in the brain of *CF1* mice by Rodriguez et al. [25].

In a murine genetic AIP model exposed to phenobarbital, an analog of barbital, Homedan et al. [61] observed a hepatic decrease in the activities of Complexes I and III and an increase in the activities of Complexes II and IV. Our results are in concordance with these findings because we observed an increase in the activities of Complexes II and IV and a decrease in the activity of Complex I due to barbital treatment in *CF1* animals which showed no alterations in PBG-D activity. 

Diaz et al. [62] observed a differential vulnerability in the different regions of the brain when evaluating specific respiratory chain defects. These authors found that the main affected region showing a decrease in the activities of Complexes III and IV was the piriform cortex; these observations are frequently observed in neurodegenerative syndromes related to alterations in electron transport, like Alzheimer’s disease.

It is important to consider that a lot of proteins, such as cytochrome P-450 (CYP), various respiratory complexes, as well as nitric oxide synthase (NOS), need heme as a prosthetic group, increasing the demand of this compound [63,64,65,66]. Low heme levels induce ALA-S, the first and regulatory enzyme, thus increasing ALA levels, which increase cellular oxidative stress [10,11] and the response of the antioxidant system. This results in the deregulation of heme synthesis because more heme is needed to satisfy its demand for CYP synthesis [67]. All the drugs studied in this work are metabolized through the Phase I drug metabolizing systemby means of CYP [30,31]. CYP2E1 is mainly responsible for the biotransformation of sevoflurane and isoflurane [68,69].

Moreover, if the respiratory chain is altered, electron transport is also altered, leading to the induction of oxidative stress, which can in turn affect respiratory complexes, membrane lipids, and therefore mitochondrial integrity. These facts could also alter the Krebs cycle, which could affect heme synthesis because the acetyl CoA generated by Krebs is a substrate of ALA-S [61,70]. Moreover, nitric oxide (NO) metabolism regulates Complex IV and a high concentration of NO leads to nitrosative stress, requiring antioxidants to protect the cell [71].

**Figure 6:**
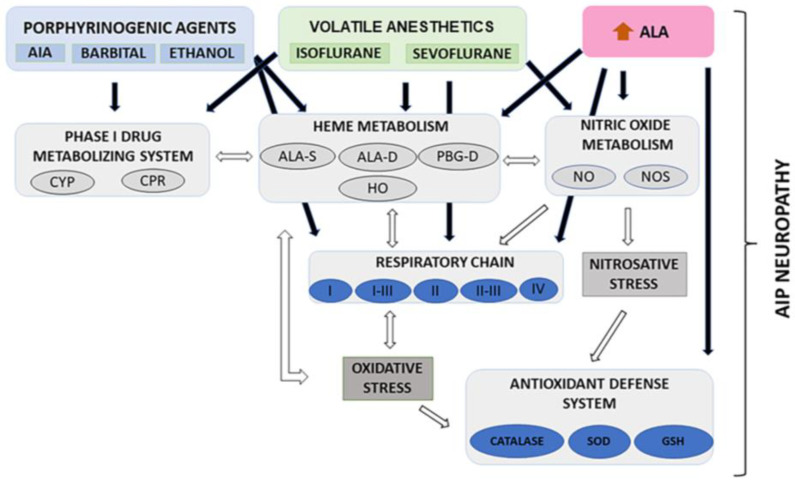
Integration of all metabolisms affected by anesthetics and other porphyrinogenic agents: allylisopropylamide (AIA), barbital and ethanol. CYP: cytochrome P-450, CPR: cytochrome P-450 reductase, ALA: 5-aminolevulinic acid, ALA-S: 5-aminolevulinic acid synthase, ALA-D: 5-aminolevulinic acid dehydratase, PBG-D: porphobilinogen deaminase, HO: heme oxygenase, NO: nitric oxide, NOS: nitric oxide synthase, SOD: superoxide dismutase, GSH: reduced glutathione, AIP: acute intermittent porphyria.

Figure 6 shows the results presented here in conjunction with our previous findings [25,30,31]. An analysis of all of them together shows that it is difficult to assign a single component to AIP neuropathy. Porphyrinogenic drugs affected heme synthesis, acting either directly on PBG-D activity or inducing ALA-S because of a reduction in the heme regulatory pool. This reduction could also be because the drugs studied are metabolized through the Phase I metabolizing system. The decrease in the heme pool affects the levels of hemeproteins such as NOS, CYP, and cytochrome c. Moreover, oxidative stress was installed due to the alterations in the antioxidant defense system reported earlier [25], which, added to NOS alterations, lead to ROS and reactive nitrogen species, affecting enzymes that contain iron–sulfur clusters such as Complexes I and III, which are targets for ROS. The links among all the metabolisms mentioned could contribute to the pathophysiology of AIP.

Previous studies attempting to explain the physiopathology of AIP have proposed that a reduction in the heme NOS cofactor could be involved [72]. NOS is also a modulator of the function of mitochondria through the interaction with Complex IV [66] and Complex I [71,72]. As it was mentioned previously, we described alterations in NOS activity and protein expression in the brains of *CF1* mice with the administration of porphyrinogenic drugs depending on the drug analyzed [32]. Moreover, it was also demonstrated that some of the porhyrinogenic agents studied would provoke a status of oxidative stress in the brain reflected by heme oxygenase alterations [25], a key enzyme of heme metabolism, which can be considered as one of the factors triggering the porphyria.

This work provides a new link in the cascade of affected metabolisms by volatile anesthetics and other porphyrinogenic drugs that involve the mitochondria and chain respiratory complexes in the brain. A differential response was found when we compared the effects of isoflurane and sevoflurane. One explanation for this behavior could be related to their metabolism through CYP, as we previously observed [24,25,30,31].

Moreover, the importance of the data described here is reinforced by the poor knowledge on the mechanisms related to the onset of AIP brain acute attacks. A better understanding of the broad action of anesthetics and other porphyrinogenic drugs altering several metabolic pathways besides the heme pathway would be crucial in translational medicine to allow us to extrapolate the results from animals to humans.

## Figures and Tables

**Table 1:** Relationship between the activities of Complexes I–III/II–III.

Treatment	Group	SP. ACT I–III/SP. ACT. II–III
Isoflurane	Control	4.03 ± 0.99
	Treated	2.14 ± 0.17
Sevoflurane	Control	9.06 ± 1.95
	Treated	5.23 ± 0.87
Allylisopropilacetamide	Control	5.38 ± 0.78
	Treated	3.90 ± 0.26
Barbital	Control	2.41 ± 0.21
	Treated	1.82 ± 0.31
Ethanol	Control	10.05 ± 0.26
	Treated	13.40 ± 1.23

## Data Availability

Data is contained within the article.
